# Evaluation of a Walking-Track Intervention to Increase Children’s Physical Activity during Primary School Break Times

**DOI:** 10.3390/children5100135

**Published:** 2018-09-25

**Authors:** Emma Powell, Lorayne A. Woodfield, Alexander J. Powell, Alan M. Nevill, Tony D. Myers

**Affiliations:** 1 Faculty of Education, Newman University Birmingham, Birmingham B32 3NT, UK; 2 Faculty of Arts, Society and Professional Studies, Newman University Birmingham, Birmingham B32 3NT, UK; l.a.woodfield@staff.newman.ac.uk (L.A.W.); alexander.powell@newman.ac.uk (A.J.P.); tony.myers@newman.ac.uk (T.D.M.); 3 Faculty of Education, Health and Wellbeing, University of Wolverhampton, Wolverhampton WV1 1LY, UK; a.m.nevill@wlv.ac.uk

**Keywords:** children, break times, physical activity, intervention, primary school

## Abstract

Despite the known benefits of engaging in daily moderate to vigorous physical activity (MVPA), only 22% of children in England are meeting the recommended guidelines. School break times have been advocated as a key part of children’s daily routines in which their MVPA can be increased. The main aim of this study was to evaluate the effect of installing a walking-track on children’s MVPA during school break times. A mixed method design was employed which allowed for the quantitative measurement of children’s PA at three time points (baseline, mid-intervention (1–5 weeks) and follow-up (6–9 weeks)), using pedometers (*n* = 81, 5–9 years) and systematic observation (*n* = 23, 7–9 years). A semi-structured interview (*n* = 1) was also conducted at 10 weeks’ follow-up. The installation of the walking-track was grounded in a unique set of theoretical constructs to aid the behaviour change of the teachers. Short term positive increases in girls’ and boys’ MVPA and longer term increases in boys’ vigorous PA (VPA) were found. Qualitative data highlighted that boys dominated the walking-track and the inconsistent behaviour of school staff negatively impacted upon children’s MVPA. A set of principles to guide the installment of walking-tracks in school playgrounds are recommended.

## 1. Introduction

For children and young people engaging in their recommended daily physical activity (PA) guidelines of at least 60 min of moderate to vigorous PA (MVPA) [1], is fundamental to both their physical and social health [2]. Despite these known benefits, only 22% of children (aged 5–15 years old) in England are meeting their recommended daily guidelines for MVPA [3]. Therefore, there is a need for MVPA to be integrated into children’s daily life [4] and the school setting is considered as central to this [5]. However, evidence suggests that children spend a large proportion of the school day engaged in sedentary activities [6,7], and the importance of reallocating children’s sedentary time (ST) and light PA (LPA) to MVPA during the school day is considered essential in order to avoid negative effects on their adiposity and cardiorespiratory fitness [8]. A multi-component approach to increasing children’s PA during the school day has been advocated which utilizes key windows of opportunity such as physical education, PA before and after school, break times, and PA during curriculum time [9]. Therefore, when considering a multi-component approach, break times can play a crucial part in contributing to children’s accumulation of their recommended MVPA; which have been defined as the non-curricular time in which children can freely engage in PA [10].

Although a growing body of research into school break times and children’s PA behaviours has accumulated over the past 20 years, systematic reviews have identified both promising strategies and inconclusive results, along with some interventions having negative effects on children’s PA [10,11]. Future research is recommended that considers both the social and physical environment of break times [11], as considering the social environment could help to develop positive attitudes in children towards PA [12]. When considering the social environment of the primary school playground, research indicates that boys and girls differ in terms of their PA behaviours, with boys enjoying sporting activities in large groups and girls preferring to socialise with their friends in small groups [12]. Furthermore, studies have evidenced that boys are more active than girls [13], and researchers have advocated for break time interventions that target these differences in children’s PA behaviours [14].

One particular intervention strategy that is popular in the UK during the primary school day is encouraging all children to walk or run a mile a day [15]. However, despite over 2000 primary schools in England taking part [15], only one study has measured the effects of such an intervention [16], which did report positive results of a 9.1 min increase in children’s daily MVPA. However, critics of such an intervention have expressed that it fails to address what children want from PA [17]. Specifying their concerns over forcing every child to run or walk a mile every day which could result in negative attitudes being developed towards PA [17]. Thus, due to the increase in schools promoting walking/running activities, it is important to gather research on the effectiveness of such interventions. Specifically, there is a lack of evidence to support walking/running activities as effective interventions during school break times.

In the design of school break time interventions to increase children’s MVPA, it is important to consider a range of factors, as highlighted by the UK’s National Institute for Health and Clinical Excellence (NICE) in their guidelines for PA intervention work. With one of their key messages being that behaviour is influenced by a number of factors including: socio-economic, cultural, environmental, social, community, and individual [18]. In addition, the guidelines also advised the use of behaviour change techniques and grounding interventions in a theoretical construct in order to create effective PA interventions [18]; suggesting that a combination of theory/models and behaviour change techniques could be the key to creating and sustaining changes in children’s PA behaviours. Thus, the use of a social ecological approach would allow researchers to consider multiple levels of influence and therefore target components of an environment for change [19]. For example, the individual environment could be targeted by catering for children’s individual interests in particular activities and alongside this, a physical change could be made to the playground environment such as implementing fixed equipment or walking tracks. 

Alongside an ecological model, it is also essential to develop a common language for the ingredients used in an intervention and the behaviour change taxonomy (BCT) [20] provides a platform for this. Furthermore, it is important to motivate school staff to change their behaviour, which includes targeting key individuals such as the head teacher, the school-level lead for the intervention and staff who will be supervising break times. Self-determination theory (SDT) has frequently been applied to PA interventions in order to motivate people to change their behaviours, as it is believed that addressing the three inner psychological needs (competency, relatedness, and autonomy) can assist in maintaining behaviour changes [21,22]. Consequently, a combination of an ecological approach, BCT ingredients and SDT could provide a successful framework for school break time interventions in order to promote positive changes and attitudes towards children’s MVPA.

This present study was part of a larger research project, in which the first stage of the research was to investigate children’s MVPA and social behaviours during primary school break times [12]. As a result of the findings from stage one, an intervention was designed to target children’s break time social behaviours. Thus, the aim of this present study was to evaluate the effect of installing a walking-track on children’s MVPA during primary school break times. The intervention was designed to encourage girls to ‘walk and talk’ around the track, as previous findings indicated that girls liked to socialise in small groups [12]. The idea being that this would provide space for the boys to engage in sporting activities which was also a previous finding from stage one of the research [12]. However, all children had a choice and could partake in any playground activity. A secondary research aim was to evaluate the effectiveness of the installation of the walking-track through exploring the school-level intervention lead’s perceptions and experiences.

## 2. Materials and Method

### 2.1. Research Design

The study was underpinned by a pragmatic philosophical approach [23], in which an explanatory mixed method design was employed [24]. The intervention design was a one-group time series, involving one experimental group which drew upon multiple points of measurements (baseline, mid-intervention (1–5 weeks), and follow-up (6–9 weeks)) [25]. Ethical approval for the study was sought and gained from the Research Ethics Committee of Newman University (project no. 2015-02-05-0000007/1569). A single school was selected due to the multiple points of measurement which enabled the participants to become their own controls, which can assist in reducing any reactivity and thus increases the reliability of the data [25].

### 2.2. Participants

In March, 2014 one primary school was selected through convenience and purposive sampling as the school expressed their aim to improve their children’s PA behaviours. The school was located in an area of high deprivation, in the West Midlands, England with 275 children on role. In January 2016, a total of 81 children (boys = 43; girls = 38) were selected (as these were the children for whom informed consent was obtained from their parents/guardians) across year groups 1, 2, 3, and 4 (aged 5 to 9 years).

### 2.3. Description of Intervention

The intervention was a physical change to the existing break time environment and involved the implementation of a 250 m long and 1 m wide track around the edge of the school field costing approximately £14,000. The track was designed so that the children could access it from the tarmac playground and as a result children’s use of the track would not be affected by weather conditions. Alongside this physical change, the social environment of break times was also targeted through providing opportunities for girls to walk and talk around the track (instead of sitting/standing and talking with their friends), whilst leaving space for boys to play sport. However, no child was forced to take part in either activity and all children could take part in any activity provided.

**Figure 1:**
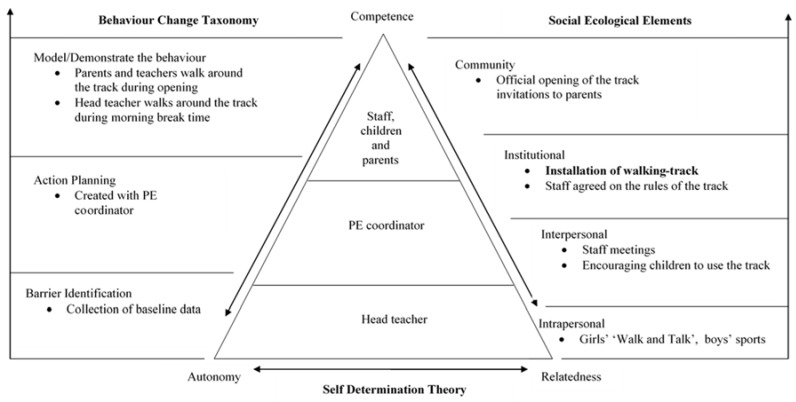
Walking-track intervention model.

As previously advocated [18], the physical change to the environment (walking-track) was grounded in a unique combination of theoretical constructs, drawing upon elements from an ecological model [19] integrating SDT [22] and key ingredients from the BCT [26]. This triangular model reflects the importance of the head teacher’s support at the base of the triangle, as without their support it was anticipated that the intervention would not be successfully implemented (Figure 1). This was then followed by the role of school-level lead for the intervention and then the roles of other school staff, the children, and their parents. To interlink these various roles within a primary school setting, SDT was applied (Table 1). Along with SDT, the intervention was grounded in four levels of the ecological health promotion model [19] (Table 1). Furthermore, ingredients from the BCT were applied (Table 1). The school was guided in the implementation of the walking track through the lead researcher meeting with the school-level lead several times in which the theoretical components of the intervention (Figure 1) were discussed and guidance was provided on how they could be implemented and managed. These meetings were then followed up with additional support e.g., email contact and telephone conversations.

### 2.4. Intervention Timeline

Initial recruitment of the school took place in March 2014 and data collection commenced in January 2016. This delay in time was due to the planning and installation of the walking track. The data collection followed three phases, the first phase of baseline data collection began in January, 2016, the second stage of mid-intervention data collection took place in February, 2016 (1–5 weeks), and the third phase of follow-up data collection commenced in April 2016 (6–9 weeks). The evaluation interview with the school level lead for the intervention took place in July 2016.

### 2.5. Setting

The pre-intervention break time setting included two tarmac playgrounds, one for Y1/Y2 (aged 5–7 years) (846.68 m^2^) and one for the Y3/Y4 (aged 7–9 years) (1311.19 m^2^). Each playground had various faded line markings such as hop scotch and snakes. The Y3/Y4 playground included rubber tires, a trim trail, basketball rings, seating huts, and the children had access to a range of portable equipment including balls, scooters, and skipping ropes. The Y1/Y2 children had access to various portable equipment, some fixed wooden climbing equipment and seating areas. Each playground was supervised during morning break time by at least two members of staff and was approximately 15 min for all year groups.

### 2.6. Measures

#### 2.6.1. Pedometers

All participants were asked to wear a sealed Yamax Digi-Walker CW700 pedometer on the right side of their hip for four consecutive days [27] during morning break time at baseline, post-intervention, and follow-up. The Yamax Digi-Walker 700/701 pedometer has been stated as being accurate and reliable in measuring step counts [28]. Pedometers are also user friendly [29], reliable [30], and have been highlighted as a useful tool for measuring changes in children’s PA [31]. Prior to use, pedometers were checked for battery life and each participant’s stride length and weight measures were entered into their personalized pedometer. All participants were provided with a pedometer step recording form, on which their class teachers recorded the number of steps on the pedometer immediately before and after morning break time.

#### 2.6.2. Anthropometric Measurements and Stride Length

Participants’ stride length was determined by each child walking 10 steps. In addition, participants’ body weight was measured to the nearest 100 g using Seca weight scales. Children’s height was measured to the nearest 0.5 cm using a Seca portable height measure. Stride length, weight, and height measures were taken within two weeks of the baseline pedometer data being collected. Child weight status was categorised [32].

#### 2.6.3. Systematic Observation: System for Observing Children’s Physical Activity and Relationships during Play (SOCARP)

The SOCARP tool was employed to provide additional insights into the PA behaviours of the children at morning break times, which assisted in identifying whether the children were using the walking track. The SOCARP tool uses systematic observation to provide data across a number of variables including: children’s activity levels, group size, activity type, and social interactions. The activity levels category is split into sedentary (defined by combining the posture codes of lying, sitting, and standing), MVPA (the sum of the walking and very active codes), and VPA (very active code). The group size category was determined by the sum of children in the group in which the observed child was located and included: alone, small (2–4 children), medium (5–9 children), and large (10+ children). Activity types were classified as: sports (e.g., an activity that was a modification of a sport with or without its official structure e.g., basketball, football, netball), active games (e.g., a non-sport game such as chase, imaginary play, dance, skipping, rough and tumble), sedentary behaviour (e.g., reading, sitting/standing talking to friends), and locomotion (e.g., travelling from A to B that is not part of a sport or active game). The social interactions category included: pro-physical, pro-verbal, anti-physical, and anti-verbal. Observers positioned themselves a maximum of 10 m away from an observed child, which enabled them to be close enough to code the social interactions category but also far enough away to reduce observer reactivity. 

For mid-intervention and follow-up data collection points, the SOCARP tool was adapted by adding the additional variable of ‘Track’ (T) to the activity category column. A small sample of Y3/4 participants (*n* = 23 (boys = 12, girls = 11)) from the 81 children wearing pedometers were each systematically observed for a 10 minute period at morning break times. This was a purposeful sample, with the criterion of a mixture of boys and girls, and who represented diversity in activity behaviours. Observations took place over a four-day period when the children were wearing the pedometers. On each observation day, two or three trained observers arrived at the school prior to morning break time. Training of observers took between 20–25 h to meet acceptable inter-observer reliability scores of >80% in each of the SOCARP categories [33]. Training was delivered by the lead researcher (EP) and involved: memorizing the SOCARP categories and codes, practicing using video examples, and also infield practice took place. SOCARP has a positive degree of content validity, and full details of the tool can be found elsewhere [33]. In addition, a field inter-observer reliability check took place with one of the observers (who was randomly selected) coding against the lead observer. The field reliability scores were >80% for each category.

#### 2.6.4. Semi-Structured Individual Interview

An in-depth individual interview was conducted with the school-level lead for the intervention after the walking-track had been implemented to evaluate the process measures of the intervention. This was considered an important evaluation tool as it was from the point of view of the person who had significant responsibility for promoting PA across the school and for the implementation of the walking-track. The interview lasted 30–40 min and a Dictaphone was used to record the verbal interactions. As a semi-structured interview guide was created in advance of the interview (informed by the quantitative results and accompanying field notes), this allowed the researcher to adapt the questions in response to the answers provided which is one of the advantages of adopting such an approach [34]. The quality of the interview data was increased through member reflections [35] during the interview process and the researcher discussed their assumptions with critical colleagues post interview [36].

### 2.7. Data Analysis

#### 2.7.1. Pedometers and SOCARP

Descriptive statistics (M ± SD) were calculated to describe the anthropometric characteristics of the children along with their step count and the % of time spent in the SOCARP categories. For the two sets of data, factorial research designs were employed to determine any main effects and interaction effects between the independent variable/s on the dependent variable/s [37]. Specifically, an independent factorial design was used for both the pedometer and SOCARP data sets, as each data set had two or more independent variables. Thus, children were treated as different participants (despite the majority of the same children being observed at each data point, some children choose not to take part or were absent for the follow-up observations). For the pedometer data, a three-way ANOVA was selected as it took into account the effect of the three independent variables (‘time’, ‘sex’, and ‘year group’) on the dependent variable of ‘mean daily morning break time step count’. For the SOCARP data, a two-way ANOVA was used to determine the effect of the two independent variables (‘time’ and ‘sex’) on the dependent variable of ‘%MVPA’ during morning break time. The interpretation of the interaction effect size was calculated using partial eta squared (ɳ_p_^2^) (small (0.01), medium (0.06) and large (0.14)) [25,38]. Bronferroni post hoc analyses were conducted to determine differences between data collection points. All statistical analyses were conducted using the Statistical Package for the Social Sciences v.23, with the alpha level being set at *p* < 0.05. The statistical assumptions for a factorial ANOVA were adhered to which included: using Levene’s test to check for equality of variances of all data points of the dependent variable and ensuring normality of residuals through the use of a QQ Plot [39].

#### 2.7.2. Semi-Structured Interview

The interview data were analysed using interpretative phenomenological analysis (IPA) [40], as the researchers were concerned with the lived experience of the school-level lead in relation to the implementation of the intervention, working within the theoretical model (Figure 1). A systematic analysis of the transcript took place in which the first step was to read and re-read the transcript, with initial notes being made. In the second step exploratory comments were produced and broken down into: descriptive (e.g., a description of the content), linguistic (e.g., specific use of language), and conceptual (e.g., an interrogation and interpretation) [40]. The third step led to the development of emergent themes; here the focus was placed upon reducing the large amount of data to discrete phrases representing the large data set. The next stage of the analysis progressed onto the abstraction of themes, at this point the themes were drawn together and a structure was produced providing organization to the analysis.

## 3. Results

### 3.1. Anthropometric Measurements

72.8% of participants were of a normal weight, with 16.0% of children being classified as overweight and 3.7% obese. In addition, 7.4% of children were underweight highlighting that more children were in the ‘underweight’ category than the ‘obese’ category.

### 3.2. Outcome Measures: Pedometers

Children’s mean pedometer steps at baseline, post-intervention and follow-up data collection points are presented in Table 2. The results revealed a main effect for ‘point of data collection’ (pre intervention, post-intervention, and follow-up) on the ‘mean pedometer steps taken during morning break time’ (*F*[2,215] = 16.22, *p* < 0.001, ɳ_p_^2^ = 0.131), accounting for 13% of the variance in children’s mean pedometers steps. The post hoc tests revealed that there was a statistically significant increase in children’s pedometer steps from baseline to post-intervention (*MD* = 236.52, *p* < 0.001, 95% CI [236.52, 351.87]). However, this was not sustained, as there was also a statistically significant decrease from post-intervention to follow-up data collection point (*MD* = −230.04, *p* < 0.001, 95% CI [−351.96, −108.11]). The results also indicated a main effect of ‘sex’ on the number of ‘mean pedometer steps taken at morning break time’ (*F*[1,215] = 21.57, *p* < 0.001, ɳ_p_^2^ = 0.091) indicating that boys accumulated more steps than girls at all three data collection points, with 9% of the variation in mean pedometer steps taken being explained by sex.

### 3.3. Outcome Measures: SOCARP

The mean (*M* ± *SD*) percent (%) of time that Y3/Y4 children spent in the SOCARP activity variables during morning break time at all three data collection points is presented in Table 3 and Table 4. The two-way ANOVA results for the SOCARP data indicated a statistically significant main effect of ‘point of data collection’ on ‘mean %MVPA’ (*F*(2,46) = 3.88, *p* = 0.028, ɳ_p_^2^ = 0.144). The post hoc test revealed a statistically significant increase in MVPA from 63.49% (9.5 min) at baseline, to 78.08% (11.7 min) at post-intervention (*MD* = 14.58 (2.2 min increase), *p* = 0.019, 95% CI (1.89, 27.28)); however the post hoc test also indicated a decrease in MVPA from post-intervention 78.08% to 72.37% (0.9 min decrease) at follow-up observation, although this decrease was not statistically significant. There were no statistically significant main effects of sex on Y3 and Y4 %MVPA at all three observations points.

The ANOVA results also revealed a main effect of ‘point of data collection’ on both boys’ and girls’ %VPA (*F*(2,46) = 6.00, *p* = 0.005, ɳ_p_^2^ = 0.207). The post hoc indicated a statistically significant increase in %VPA from baseline to post-intervention (*MD* = 15.43 (2.3 min increase), *p* = 0.01, 95% CI (2.91–27.96)) and this was maintained at follow-up observation (*MD* = 14.04 (2.1 min increase), *p* = 0.02, 95% CI (1.25–26.84)). Although there were no statistically significant main effects of ‘sex’ on %VPA; boys’ %VPA increased at all three time points (baseline to follow-up data, *MD* = 16.38 (2.5 min increase), *p* = 0.06, 95% CI (−0.64–33.39)), whereas girls’ %VPA increased at post-intervention (2.5 min increase) but then declined at follow-up data collection point (0.7 min decrease) (Table 3).

Time (%) spent using the walking-track declined between post-intervention and follow-up data collection observations (*MD* = −34.90, *p* = 0.002, 95% CI (−60.09, −9.70)), with a main effect for ‘point of data collection’ (pre-intervention, post-intervention, and follow-up) on ‘% of time spent using the track’ (*F*(2,46) = 17.27, *p =* 0.004, ɳ_p_^2^ = 0.429). The results also highlighted a statistically significant interaction effect between ‘point of data collection’ and ‘sex’ on the ‘% of time children spent engaged in sports activities’ during break time (*F*(2,46) = 5.48, *p* = 0.007, ɳ_p_^2^ = 0.192). This signifies that the effect of ‘point of data collection’ on children’s engagement in sports activities differed for boys and girls, with boys engaging in more sports activities than girls. This suggests that the effect of the intervention differed between sexes.

### 3.4. Process Measure: Individual Interview

From analysis of the interview data, the following themes emerged: boys’ domination of the walking track, and conflicting visions of school staff.

Boys’ domination of the walking-track (emergent themes: racing games; imaginary play; and girls sitting and talking). The interview data revealed that the boys’ dominated the walking-track during morning break time. The intervention lead reported that the boys enjoyed racing around the track, being timed by the teachers and they also engaged in imaginary play behaviour on the track. The intervention lead described how the boys used the track more than the girls; although the girls did use the track, the interview data highlighted that the girls would often sit or stand chatting away from the walking track. For instance:

- “The boys bound on past the girls who are walking by, they might intimidate them a little bit but yeah the boys seem to access it more.”
- “Boys like playing superheroes around the track and pretending they are superman.”
- “They [the girls] go off by the huts and kind of lean on the huts and have a chat; they go on the benches and continue their chats on there.”

Conflicting visions of school staff (emergent themes: lack of buy in from school staff; and not every child wants to be active). During the interview the intervention lead revealed how some of the senior members of staff wanted to offer children the opportunity to write or draw during break times instead of walking around the track. The intervention lead also discussed how benches had been placed around the track, but they did not understand the reasoning behind this decision. As illustrated in the following quotes:

- “It was felt that there was a lacking in [sic] creative things for the children who would like to be drawing and writing so some staff thought it would be a good idea to take chalk out and that’s how it came about really, so they kind of put it out there and we had to agree really so that’s how it came about.”
- “I can’t really explain the benches, I think some staff thought it would be nice as a scenic, you know, sitting and chatting space which isn’t what we were aiming for really but I guess they are thinking for those that don’t want to.”

## 4. Discussion

The main aim of this study was to evaluate the effect of installing a walking-track on children’s MVPA during primary school break times. A secondary research aim was to evaluate the effectiveness of the installation of the walking-track through exploring the school-level intervention lead’s perceptions and experiences. The results evidenced that the walking-track intervention had a positive short-term effect on children’s step count during morning break time (236 step increase at mid intervention) however; this was not maintained at the 6–9 weeks follow-up and returned to baseline figures. Likewise, the initial percentage point increase in MVPA (14.58% increase from baseline to mid-intervention) was not sustained at follow-up (8.88% point increase from baseline). Though, an interesting finding was the increase in boys’ %VPA at mid-intervention which then continued to increase at follow-up data collection (16.38% point increase).

From the interview findings, it could be suggested that boys’ utilised the track in an imaginative way. As has been previously suggested children are resourceful with their environment and often engage in imaginative play [12]. Additionally, previous research [41,42] and the findings from this study suggest that boys often dominate the playground space. The baseline data in this study suggests that boys initially dominated the playground playing sports, engaging in a mean of 40.67% in sports activities during morning break times. However, at mid-intervention (1–5 weeks) and follow-up data collection (6–9 weeks), the SOCARP data indicated that none of the observed boys engaged in any sports activities. Furthermore, the qualitative data indicated that boys dominated the walking-track playing racing games and superheroes. Thus, it can be suggested that the walking-track had a more positive effect on boys’ PA, but this negatively impacted upon the girls’ PA due to the boys’ domination of the track, which was not an intention of the intervention. Thus, a suggestion for future studies is to provide different areas or time periods for boys and girls in which they can access a walking track.

Another finding from this study was the inconsistencies and the practice of some school staff during the intervention; as highlighted in the qualitative data, some teachers were concerned that children needed other options at break times. From the field notes, which were taken during the employment of the SOCARP tool, researchers stated that benches had been placed around the edge of the walking-track and some teachers had provided children with chalk, which seemed particularly popular with the girls during break times. The interview data revealed that the intervention lead did not agree with these inconsistencies as they did not align with the vision for increasing children’s PA at break times. Accordingly, the implementation of the benches and having chalk during break times could have encouraged some children to engage in sedentary and low intensity activity and therefore, may explain the follow-up data collection results.

Furthermore, at the time of the intervention the school introduced ‘The Daily Mile’ [15] activity during curriculum time for all year groups. As a result, children were increasing their PA levels at other times in the school day, which could have had a negative effect on their break time PA behaviour. There is limited research on children’s PA compensation during the school day [43], however, it has been suggested that children will compensate for high amounts of PA participation by lowering energy expenditure at a later time [44]. Thus, although initial research indicates positive increases from ‘The Daily Mile’ [16], it may be at the detriment of children’s PA during key windows of opportunity in the school day such as break times. Consequently, further research into the compensation of children’s PA during the school day when introducing ‘The Daily Mile’ in curriculum time is warranted. Furthermore, when implementing an intervention in one segment of the school day—e.g., break times, active lessons, PE lessons, etc.—it would be beneficial to also use accelerometers to measure PA across the school day which would help to identify any PA compensation. 

The implementation of ‘The Daily Mile’, alongside resources that promoted sedentary behaviours amongst the children were not advised by the researchers, who had no control over these additional playground features and curriculum time initiatives. These observations are consistent with other break time intervention studies which have reported inconsistencies from teachers in the implementation of interventions [45]. The inconsistencies in the implementation and use of the walking-track are something that needs to be taken into consideration in the design of future break time PA interventions; which could be achieved through the careful selection of key ingredients from the BCT [20].

As advocated by NICE [18], PA interventions need to be grounded in both behaviour change theory and theoretical frameworks. Thus, it is important to evaluate the effectiveness of such frameworks. The break time intervention model (Figure 1), had integrated the SDT [22], elements from the ecological model for health promotion [19], and three key ingredients from the BCT [20] (Figure 1). When taking into consideration the BCT ingredients and the inconsistencies in the implementation of the intervention from school staff, the additional BCT key ingredient of ‘provide instruction’ may have been beneficial in overcoming this limitation so that the intervention could have been consistently implemented across the school. This technique has been described as instructing people in ‘how to’ do something rather than ‘what to do’ [20]. Accordingly, a recommendation for future break time interventions to increase children’s PA would be to adapt ‘the walking-track intervention model’ (Figure 1) to include the BCT ingredient of ‘provide instruction’.

## 5. Limitations

One limitation of the study was having no control group to compare the intervention effects against. Nonetheless, the application of a time series design can allow the participants to act as their own control group [25]. However, implementing the intervention in and collecting data in one school does limit the generalizability of the study’s findings to other school contexts. Moreover, resource limitations led to a small sample being observed when employing the SOCARP tool. The intense nature of this tool is expensive in terms of researcher time. However, observation was employed alongside the pedometers and it did provide an additional insight into children’s break time PA behaviour. It is also acknowledged that the presence of the researchers during break times could have influenced the children’s PA behaviours [34]. Furthermore, the parallel intervention of the Daily Mile implemented by the school could have impacted upon the results of the walking-track intervention. Moreover, the measurement of children’s PA during lunch times was not included as the school wanted to initially pilot the use of the walking track at morning break times. 

## 6. Conclusions

The walking-track intervention was designed to increase children’s MVPA levels during primary school break times. The results indicate that the intervention did have positive short-term effects (1–5 weeks), in relation to both boys’ and girls’ step count and %MVPA, and longer positive effects (6–9 week) in relation to boys’ %VPA. Thus, it is suggested that the implementation of a walking-track in the grounds of a primary school can have positive longer-term effects on boys’ VPA, which could contribute to them achieving their daily PA recommendations of at least 60 min MVPA. However, the inconsistencies in the implementation and use of the track are something that needs to be taken into consideration as these impacted upon the results of the study and girls’ PA levels. As such, a future recommendation would be to test the effectiveness of the walking-track intervention which has integrated the additional BCT ingredient of ‘provide instruction’ [20] through the creation and communication of a set of ‘how to’ principles.

## Figures and Tables

**Table 1:** Walking-track intervention model theoretical components.

Social Ecological Components [19]	Behaviour Change Taxonomy [20]	Self Determination Theory [22]
**Intrapersonal**	**Barrier Identification/Problem Solving**	**Competence**
The track was aimed at encouraging girls to ‘walk and talk’ during morning break times, without impeding upon the playground space of boys’ sporting activities such as football.	An initial decision to target children’s PA behaviour from the intervention lead and head teacher. Collection of baseline data provided an understanding of children’s current PA levels during morning break times. Baseline data included children’s pedometer step counts and SOCARP data. Meetings between the researcher and intervention lead to identify possible ways to address low levels of MVPA during break times, especially in relation to girls’ PA behaviour. Discussions included implementing a walking-track on the school field.	The intervention lead’s competence developed through awareness and discussions of possible intervention strategies with the researcher. Thus, they were able to successfully lead the implementation of the walking route on the school field.
**Interpersonal**	**Action Planning**	**Relatedness**
Children’s use of the track during morning break time was discussed in a staff meeting led by the intervention lead and head teacher. The teachers agreed the school rules of the track which included all children being able to access the track at morning break times if they wished to do so (Y1, Y2, Y3, and Y4].	Creation of a school action plan for increasing children’s PA levels, within this included targets specific to morning break times. Action planning included: ‘target’, ‘rationale’, ‘action’, timescale’, and ‘evidence/outcome’. Example of the targets were ‘to increase children’s PA levels during break times’, ‘to create a walk and talk route for girls during break times’, and ‘to collect post-intervention and follow-up data to measure the sustainability of the intervention’.	From the head teacher’s and intervention lead’s support, staff, children, and their parents were aware of and had walked around the track, thus creating relatedness and a sense of belonging. Staff were involved in planning meetings and the development of the formal rules of the track.
**Institutional**	**Model/Demonstrate the Behaviour**	**Autonomy**
Implementation of a 250 m long and 1 m wide gravel walking-track around the perimeter of the school field. This could be accessed by all children.	IThis involved both parents and teachers modelling the behaviour of walking around the track for the children, which took place during the opening celebration. In addition, during morning break time the head teacher would frequently walk around the track with the children.	Children were in control of their own behaviour as they had a choice as to whether they walked around the track during morning break time. No set days were allocated for year groups, all children could access the track during morning break times. Several ideas were discussed with the school in how to change the children’s PA break time behaviour, the school decided to implement the walking-track on the school field.
**Community**		
Official opening of the track, with the children, their parents and teachers.		

**Table 2:** Children’s pedometer step count during morning break times: baseline, post-intervention, and follow-up data collection points (*M* ± *SD*).

	Baseline (*M* ± *SD*) (*n* = 81)	Post Intervention (*M* ± *SD*) (*n* = 75) (1–5 Weeks)	Follow-up (*M* ± *SD*) (*n* = 68) (6–9 Weeks)
Whole school	1176.43 ± 366.42	1412.95 ± 348.22	1182.91 ± 306.55
Boys	1235.29 ± 364.37	1495.31 ± 368.47	1293.75 ± 222.47
Girls	1096.31 ± 358.75	1336.93 ± 314.21	1050.63 ± 342.42
Y1	1125.26 ± 230.90	1437.44 ± 393.06	1194.53 ± 169.36
Y2	971.87 ± 235.41	1238.87 ± 283.755	1218.30 ± 297.61
Y3	1480.51 ± 354.05	1605.85 ± 289.64	1137.55 ± 337.56
Y4	1098.23 ± 366.42	1305.14 ± 290.63	1195.36 ± 370.39

**Table 3:** Changes in Y3 and Y4 mean (*M* ± *SD*) percentage (%) of morning break times spent in the SOCARP activity variables of: ‘activity level’ and ‘activity type’ at baseline, post-intervention, and follow-up data collection points.

	Baseline (*n* = 23)			Post-Intervention (*n* = 15) (1–2 weeks)			Follow-up (*n* = 14) (6–7 Weeks)		
	Boys (*n* = 12)	Girls (*n* = 11)	Boys and Girls (*n* = 23)	Boys (*n* = 6)	Girls (*n* = 9)	Boys and Girls (*n* = 15)	Boys (*n* = 7)	Girls (*n* = 7)	Boys and Girls (*n* = 14)
**Activity Level**									
Lying down	1.39 ± 4.81	0.00 ± 0.00	0.72 ± 3.48	0.00 ± 0.00	0.00 ± 0.00	0.00 ± 0.00	1.43 ± 3.78	0.00 ± 0.00	0.71 ± 2.67
Sitting	3.97 ± 5.91	4.09 ± 7.94	4.03 ± 6.79	4.39 ± 7.27	0.37 ± 1.11	1.98 ± 4.87	6.95 ± 11.18	5.07 ± 12.00	6.01 ± 11.19
Standing	33.06 ± 23.65	31.61 ± 10.98	32.36 ± 18.30	16.99 ± 19.29	18.33 ± 17.43	17.80 ± 17.52	17.54 ± 5.75	22.72 ± 9.68	20.13 ± 8.12
Walking	42.79 ± 16.52	49.53 ± 11.93	46.01 ± 14.60	45.49 ± 20.78	44.94 ± 18.45	45.16 ± 18.68	37.63 ± 11.92	44.06 ± 14.46	40.84 ± 13.16
Vigorous	18.52 ± 13.58	16.35 ± 14.12	17.48 ± 13.57	32.02 ± 16.55	33.40 ± 18.19	32.92 ± 16.95	34.90 ± 11.53	28.16 ± 17.22	31.53 ± 14.50
Sedentary	38.42 ± 22.75	35.70 ± 9.67	37.12 ± 17.41	21.38 ± 17.91	18.70 ± 17.26	19.78 ± 16.66	25.92 ± 14.35	27.78 ± 11.87	26.85 ± 12.69
MVPA	61.30 ± 22.44	65.89 ± 6.58	63.49 ± 16.64	77.69 ± 17.03	78.33 ± 16.57	78.08 ± 16.14	72.53 ± 13.46	72.22 ± 11.87	72.37 ± 12.19
**Activity Type**									
Track	0.00 ± 0.00	0.00 ± 0.00	0.00 ± 0.00	56.02 ± 48.06	51.61 ± 40.85	53.37 ± 42.23	22.47 ± 27.66	14.48 ± 28.15	18.48 ± 27.13
Sports	41.67 ± 47.34	0.00 ± 0.00	21.74 ± 39.67	0.00 ± 0.00	0.00 ± 0.00	0.00 ± 0.00	0.00 ± 0.00	3.43 ± 9.07	1.71 ± 6.41
Games	15.14 ± 26.06	41.03 ± 28.01	27.52 ± 29.51	7.01 ± 15.05	7.04 ± 17.52	7.03 ± 16.01	28.79 ± 31.55	19.58 ± 29.44	24.18 ± 29.70
Sedentary	27.62 ± 31.23	31.85 ± 16.77	29.64 ± 24.90	22.25 ± 18.71	13.98 ± 17.71	17.29 ± 17.94	18.45 ± 5.18	24.83 ± 12.02	21.64 ± 9.49
Locomotion	15.57 ± 23.50	25.45 ± 20.73	20.30 ± 22.29	76.42 ± 18.44	75.65 ± 27.18	75.96 ± 23.32	44.26 ± 32.52	54.93 ± 22.41	49.59 ± 27.40

**Table 4:** Changes in Y3 and Y4 mean (*M* ± *SD*) percentage (%) of morning break times spent in the SOCARP activity variables of: ‘group size’ and ‘social interactions’ at baseline, post-intervention, and follow-up data collection points.

	Baseline (*n* = 23)			Post-Intervention (*n* = 15) (1–2 weeks)			Follow-up (*n* = 14) (6–7 Weeks)		
	Boys (*n* = 12)	Girls (*n* = 11)	Boys and Girls (*n* = 23)	Boys (*n* = 6)	Girls (*n* = 9)	Boys and Girls (*n* = 15)	Boys (*n* = 7)	Girls (*n* = 7)	Boys and Girls (*n* = 14)
**Group Size**									
Alone	9.20 ± 11.65	12.20 ± 14.19	10.63 ± 12.72	47.20 ± 21.58	20.83 ± 18.07	31.38 ± 23.06	19.51 ± 17.77	8.80 ± 7.27	14.16 ± 14.18
Small	47.58 ± 38.48	69.03 ± 26.62	57.84 ± 34.39	43.77 ± 28.89	56.76 ± 27.27	51.56 ± 27.69	64.00 ± 32.59	76.41 ± 30.25	70.21 ± 30.89
Medium	26.56 ± 39.11	17.50 ± 28.47	22.23 ± 33.98	2.841 ± 5.07	18.06 ± 26.57	11.97 ± 21.73	5.26 ± 8.00	7.29 ± 9.87	6.28 ± 8.69
Large	16.67 ± 38.92	0.30 ± 1.01	8.84 ± 28.77	7.64 ± 18.71	1.39 ± 4.17	3.89 ± 12.04	10.71 ± 28.35	10.56 ± 27.94	10.64 ± 27.04
**Social Interactions**									
Pro-physical	29.27 ± 27.75	20.06 ± 23.37	24.86 ± 25.60	9.52 ± 23.33	12.88 ± 13.24	11.54 ± 17.25	13.20 ± 8.82	17.74 ± 17.00	15.47 ± 13.22
Pro-verbal	63.15 ± 30.03	78.89 ± 24.38	70.68 ± 28.03	88.96 ± 22.88	86.47 ± 14.56	87.46 ± 17.60	85.65 ± 9.15	80.96 ± 19.62	83.31 ± 14.91
Anti-physical	4.70 ± 7.49	0.72 ± 2.38	2.79 ± 5.90	1.52 ± 3.71	0.65 ± 1.96	1.00 ± 2.70	1.14 ± 3.02	0.00 ± 0.00	0.57 ± 2.14
Anti-verbal	0.48 ± 1.12	0.00 ± 0.00	0.25 ± 0.83	0.00 ± 0.00	0.00 ± 0.00	0.00 ± 0.00	0.00 ± 0.00	0.00 ± 0.00	0.00 ± 0.00
